# Supplementary material for: Statistical patterns in the Ethereum blockchain: Analysis of EOAs and smart contracts in ERC20 token network
Source: PLoS One. 2026 Oct 1;21(10):e0358972. doi: 10.1371/journal.pone.0358972 (PMC13630221; doi:10.1371/journal.pone.0358972)
Supplement: Supplementary file 1 [file pone.0358972.s001.pdf]

Statistical patterns in the Ethereum blockchain: analysis of EOAs and smart contracts in ERC20 token network

Kundan Mukhia [1◕], SR Luwang [1] Md. Nurujjaman[1‡] Tanujit Chakraborty [2,3*], Suman Saha[4*], Chittaranjan Hens[5],

1 Department of Physics, National Institute of Technology, 737139, Sikkim, India
2 SAFIR, Sorbonne University, Abu Dhabi, UAE
3 Sorbonne Center for Artificial Intelligence, Sorbonne University, Paris, France
4 School of Electronics Engineering, Vellore Institute of Technology, Chennai, Tamil Nadu 600127, India
5 Centre for Computational Natural Sciences and Bioinformatics, International Institute of Information Technology, Hyderabad 500032, India.

◕kundanmukhia07@gmail.com salamrabindrajit@gmail.com
‡md.nurujjaman@nitsikkim.ac.in *tanujit.chakraborty@sorbonne.ae
*ecesuman06@gmail.com chittaranjanhens@gmail.com

1 Figure S1: Supporting Information

Figure 1: Period 4, Scatter plots in log-log scale depict the relationship between the number of trades executed per unique trader and the number of unique trade partners. Each subplot corresponds to one transaction type: (a) EOA–EOA, (b) EOA–SC, (c) SC–EOA, and (d) SC-SC. Black dots represent individual traders, while brown curves show log-binned averages, highlighting the underlying scaling trend. These visualizations provide insight into how trade activity scales against partner diversity.
