# Supplementary material for: Statistical patterns in the Ethereum blockchain: Analysis of EOAs and smart contracts in ERC20 token network
Source: PLoS One. 2026 Oct 1;21(10):e0358972. doi: 10.1371/journal.pone.0358972 (PMC13630221; doi:10.1371/journal.pone.0358972)
Supplement: Supplementary file 2 [file pone.0358972.s002.pdf]

Statistical patterns in the Ethereum blockchain: analysis of EOAs and smart contracts in ERC20 token network

Kundan Mukhia [1◕], SR Luwang [1] Md. Nurujjaman[1‡] Tanujit Chakraborty [2,3*], Suman Saha[4*], Chittaranjan Hens[5],

1 Department of Physics, National Institute of Technology, 737139, Sikkim, India
2 SAFIR, Sorbonne University, Abu Dhabi, UAE
3 Sorbonne Center for Artificial Intelligence, Sorbonne University, Paris, France
4 School of Electronics Engineering, Vellore Institute of Technology, Chennai, Tamil Nadu 600127, India
5 Centre for Computational Natural Sciences and Bioinformatics, International Institute of Information Technology, Hyderabad 500032, India.

◕kundanmukhia07@gmail.com salamrabindrajit@gmail.com
‡md.nurujjaman@nitsikkim.ac.in *tanujit.chakraborty@sorbonne.ae
*ecesuman06@gmail.com chittaranjanhens@gmail.com

1 S1 Table: Supporting Information

Table 1: S1 Table. TL exponents β for **30-minute** time window measuring the scaling relationship between the variance and the mean of trading activity across different transaction types and roles over three time periods. Values report the log–log slope (β), 95% confidence interval (CI), coefficient of determination (R^2), and number of observations (N).

Transaction	Role	Period	β	CI (95%)	R^2	N
EOA–EOA	Sender	1	2.29	[2.27, 2.31]	0.83	11,169
	Sender	2	2.27	[2.26, 2.29]	0.87	26,261
	Sender	3	2.15	[2.14, 2.16]	0.87	42,200
	Receiver	1	2.29	[2.27, 2.30]	0.75	22,060
	Receiver	2	2.32	[2.30, 2.33]	0.75	40,915
	Receiver	3	2,30	[2.29,2.31]	0.78	78,256
EOA–SC	Sender	1	2.29	[2.24, 2.34]	0.81	1,970
	Sender	2	2.22	[2.18, 2.26]	0.77	4,026
	Sender	3	2.34	[2.29, 2.38]	0.79	2,668
	Receiver	1	2.34	[2.28, 2.39]	0.87	980
	Receiver	2	2.38	[2.32, 2.44]	0.83	1,198
	Receiver	3	2.38	[2.32, 2.44]	0.83	1,198
SC–EOA	Sender	1	2.29	[2.24, 2.35]	0.90	736
	Sender	2	2.13	[2.10, 2.17]	0.93	1,318
	Sender	3	2.10	[2.07, 2.12]	0.95	1,572
	Receiver	1	2.33	[2.31, 2.35]	0.87	8,016
	Receiver	2	2.59	[2.56, 2.62]	0.86	4,771
	Receiver	3	2.34	[2.31, 2.37]	0.82	6,527
SC–SC	Sender	1	2.44	[2.31, 2.57]	0.88	190
	Sender	2	2.24	[2.13, 2.36]	0.83	325
	Sender	3	2.21	[2.11, 2.31]	0.82	393
	Receiver	1	2.24	[2.14, 2.34]	0.79	492
	Receiver	2	1.91	[1.84, 1.99]	0.88	333
	Receiver	3	2.02	[1.90, 2.14]	0.76	349
