# Supplementary material for: Statistical patterns in the Ethereum blockchain: Analysis of EOAs and smart contracts in ERC20 token network
Source: PLoS One. 2026 Oct 1;21(10):e0358972. doi: 10.1371/journal.pone.0358972 (PMC13630221; doi:10.1371/journal.pone.0358972)
Supplement: Supplementary file 3 [file pone.0358972.s003.pdf]

Statistical patterns in the Ethereum blockchain: analysis of EOAs and smart contracts in ERC20 token network

Kundan Mukhia [1◕], SR Luwang [1] Md. Nurujjaman[1‡] Tanujit Chakraborty [2,3*], Suman Saha[4*], Chittaranjan Hens[5],

1 Department of Physics, National Institute of Technology, 737139, Sikkim, India
2 SAFIR, Sorbonne University, Abu Dhabi, UAE
3 Sorbonne Center for Artificial Intelligence, Sorbonne University, Paris, France
4 School of Electronics Engineering, Vellore Institute of Technology, Chennai, Tamil Nadu 600127, India
5 Centre for Computational Natural Sciences and Bioinformatics, International Institute of Information Technology, Hyderabad 500032, India.

◕kundanmukhia07@gmail.com salamrabindrajit@gmail.com
‡md.nurujjaman@nitsikkim.ac.in *tanujit.chakraborty@sorbonne.ae
*ecesuman06@gmail.com chittaranjanhens@gmail.com

1 S2 Table: Supporting Information

Table 1: S2 Table. TL exponents β for **2-hour** time window measuring the scaling relationship between variance and mean of trading activity across different transaction types and roles over three time periods. Values report the log–log slope (β), 95% confidence interval (CI), coefficient of determination (R^2), and number of observations (N).

Transaction	Role	Period	β	CI (95%)	R^2	N
EOA–EOA	Sender	1	2.33	[2.31, 2.34]	0.85	15,724
	Sender	2	2.34	[2.33, 2.35]	0.88	35,812
	Sender	3	2.22	[2.22, 2.23]	0.87	59,514
	Receiver	1	2.39	[2.37, 2.40]	0.77	34,133
	Receiver	2	2.41	[2.40, 2.42]	0.76	59,918
	Receiver	3	2.39	[2.38, 2.40]	0.81	113,852
EOA–SC	Sender	1	2.25	[2.21, 2.29]	0.79	3,565
	Sender	2	2.29	[2.26, 2.32]	0.76	7,116
	Sender	3	2.35	[2.32, 2.38]	0.82	5,320
	Receiver	1	2.24	[2.19, 2.28]	0.83	2,088
	Receiver	2	2.32	[2.28, 2.37]	0.83	2,270
	Receiver	3	2.27	[2.24, 2.30]	0.90	2,240
SC–EOA	Sender	1	2.31	[2.27, 2.34]	0.91	1,543
	Sender	2	2.20	[2.18, 2.23]	0.94	2,189
	Sender	3	2.15	[2.13, 2.17]	0.95	2,465
	Receiver	1	2.48	[2.47, 2.50]	0.90	9,807
	Receiver	2	2.46	[2.44, 2.48]	0.86	8,610
	Receiver	3	2.40	[2.38, 2.42]	0.82	11,115
SC–SC	Sender	1	2.43	[2.32, 2.53]	0.88	286
	Sender	2	2.26	[2.17, 2.35]	0.85	423
	Sender	3	2.22	[2.14, 2.30]	0.84	500
	Receiver	1	2.12	[2.02, 2.21]	0.75	661
	Receiver	2	2.00	[1.93, 2.06]	0.90	424
	Receiver	3	2.14	[2.05, 2.23]	0.82	439
