# Supplementary material for: Statistical patterns in the Ethereum blockchain: Analysis of EOAs and smart contracts in ERC20 token network
Source: PLoS One. 2026 Oct 1;21(10):e0358972. doi: 10.1371/journal.pone.0358972 (PMC13630221; doi:10.1371/journal.pone.0358972)
Supplement: Supplementary file 4 [file pone.0358972.s005.docx]

We extend our sincere thanks to the editors and reviewers for the thoughtful suggestions and comments, which greatly helped to improve our work. We carefully addressed each of the reviewers' comments point by point. Based on their suggestions, we have made a major overhaul of the main text with particular attention to rewriting the abstract, method, and results sections for improved clarity and delineating the scientific impact of our findings. In the revised version, we have thoroughly revised and reorganized the Results section, revised figures, and reproduced results. Further to this, we have also undertaken new analysis following reviewer recommendations.

# Summary of the revisions

# In the revised manuscript, we have highlighted our revisions in red-colored text. Below, we also provide a summary of major revisions carried out in the revised version of the manuscript.

| # | Category | Changes Made |
| --- | --- | --- |
| 1 | New Additions | • We performed an out-of-sample validation on ERC20 data for Period 4: December 2019 – February 2020 to test the robustness of our results, listed in the Tables in the main text and Fig. S1 in Supporting Information.  • A new section on gas fee analysis has been included.  • Table 6 displays results on Gas fee statistics using block transaction data, period July 2017- March 2018, and December 2019-February 2020.  • Tables S1 and S2 in the Supporting Information for the analysis of time-scale sensitivity results using two other different time windows of 30 minutes and 2 hours. |
| 2 | Tables Updated | • Table 1 has been revised to include Period 4 results.  • Tables 2, 3, and 5 have been updated to include 95% confidence intervals (CI), R² values, and sample sizes (N). |
| 3 | Figures Revised | • Figures 1–3 have been updated with improved axis formatting and added confidence interval information.  • Figures 4–7 now include x_min_ and threshold annotations, along with vertical dashed lines.  • Figures 8–11 have been revised to include CI and R² values for better clarity. |
| 4 | Supporting Information | Supplementary information is added, which includes one new figure for extended period analysis, and two tables for Sensitivity analysis of Taylor’s Law using two different time windows. |
| 5 | Conceptual and Textual Revisions | • We have replaced the terms “human-driven” and “algorithm-driven” with “EOA-driven” and “SC-driven” throughout the manuscript.  • The unit of analysis has been clarified as address-level (pooled across tokens).  • Additional discussion on out-of-sample validation has been added in relevant sections.  • We have corrected an inconsistency in the SC–SC scaling exponent (0.62 revised to 0.67). |
| 6 | Reproducibility Improvements | • We have provided a public GitHub repository with complete code and documentation.  • We added a Data Availability Statement specifying the dataset source and block ranges to reproduce the analysis. |
| 7 | Title | "Statistical pattern in the Ethereum blockchain: Analysis of EOAs and smart contracts in ERC20 token network" |

# REVIEWER’S COMMENTS

REVIEWER #2

**Reviewer’s Comment : Hedge “universal” language; anchor claims to the July 2017–March 2018 window stated in the abstract and dataset description, and rephrase as period‑specific until validated.**

**Authors' Response:** We sincerely thank the reviewer for this constructive suggestion. We have hedged the term "Universal" and revised the title accordingly, from "Universal pattern in the Blockchain: Analysis of EOAs and smart contracts in ERC20 token network" to "Statistical pattern in the Ethereum blockchain: Analysis of EOAs and smart contracts in ERC20 token network" in the revised manuscript.

We have also revised the language in the abstract accordingly. Please see, Section: Abstract, Page: 1, Line: 25–26.

**Original:** "To identify universal statistical patterns, we investigate the presence of two canonical scaling laws: power law distributions and temporal Taylor's law (TL)."

**Revised:** "To characterize and identify specific statistical patterns, we investigate the presence of two canonical scaling laws: power law distributions and temporal Taylor's law (TL)."

**Reviewer’s Comment : It would be nice to add out‑of‑sample validation (more recent years and, if feasible, another chain) to test stability of the reported regularities before generalizing.**

**Authors' Response:** In the revised version, we have extended the time period from December 2019 to February 2020, and conducted the out-of-sample validation using the ERC20 transaction data. The total number of transactions in this period is 36,692,530. We applied similar analysis such as scaling law, power-law distribution, and temporal Taylor's law, to this validation dataset. We mentioned this validation period as Period 4 in our study. Details of the results are presented in the tables below. It can be concluded that the results obtained for Period 4 are consistent with Periods 1, 2, and 3, confirming that the qualitative distinction between EOA and SC behavioral signatures is robust across all four time periods.

Additionally, in the revised manuscript, we clearly mentioned in the discussion as a limitation the inability to validate results on other chains due to the unavailability of comparable datasets, and the potential for extending this work across multiple chains in future research. Please see - **Page: 23, line: 604-606.**

The following plots illustrate the results from scaling law analysis on Period 4 for EOA-EOA, EOA-SC, SC-EOA, and SC-SC transactions. We have incorporated the scaling law plot for Period 4 in the supporting information. Please see- **Section: Supporting Information, Page: 23-24, line: 616–624.**

Figure S1: “Period 4, Scatter plots in log-log scale depict the relationship between the number of trades executed per unique trader and the number of unique trade partners. Each subplot corresponds to one transaction type: (a) EOA–EOA, (b) EOA–SC, (c) SC–EOA, and (d) SC-SC. Black dots represent individual traders, while brown curves show log-binned averages, highlighting the underlying scaling trend. These visualizations provide insight into how trade activity scales against partner diversity.”

In the revised manuscript, we have incorporated the following changes:

**(a) Section: Abstract, Page: 1, line: 19–22.**

***Earlier version:*** *“*This study reveals distinctive statistical signatures in the transactional dynamics of ERC20 tokens on the Ethereum blockchain by examining over 44 million token transfers between July 2017 and March 2018 (9-month period).”

***Revised version:*** "This study reveals distinctive statistical signatures in the transactional dynamics of ERC20 tokens on the Ethereum blockchain by examining over 81 million token transfers across two independent time windows: July 2017 to March 2018 and December 2019 to February 2020."

**(b) Section: ERC20 Transaction Dataset, Page: 3-4, line: 139–145.**

"To assess the robustness of our findings, we additionally analysed an out-of-sample validation dataset comprising 36,692,530 ERC20 token transfer records from December 2019 to February 2020. This validation period captures the early DeFi ecosystem, representing fundamentally different market conditions from the primary dataset. In total, this study analyses over 81 million ERC20 token transfer records across two independent time windows."

**(c) Section: ERC20 Transaction Dataset, Page: 4, line: 162–167.**

"Additionally, Period 4 covers data from December 2019 to February 2020 and serves as an out-of-sample validation period to assess the robustness of the reported findings."

**(d) Section: ERC20 Transaction Dataset, Table 1, Page: 4, columns 7 and 8.**

***Earlier caption:*** *“*Classification of ERC20 token transactions based on the sender and receiver account types, with the transactional volume shown for the full dataset (Total Transaction), divided into three equal time periods.”

***Revised caption:*** *“*Classification of ERC20 token transactions based on the sender and receiver account types, with the transactional volume shown for the full dataset (Total Transaction), divided into four equal time periods.”

**(e) Section: Scaling Relationship between Trade Volume and Partner Diversity, Table 2, Page: 10, Column: 2-6 and 3, Row: 4, 8, 12, and 16.**

**(f) Section: Scaling Relationship between Trade Volume and Partner Diversity, Page: 9, line: 386–390.**

"These patterns are also observed in the out-of-sample validation for Period 4, where similar scaling exponents across all transaction types further confirm the robustness of the EOA and SC behavioural distinction across independent time windows as shown in S1 Fig."

**(g) Section: Power-law patterns in trading activity, Table 3, Page: 14, Column: 2-10, Row:7, 8, 15, 16, 23, 24, 31, and 32**

**(h) Section: Power-law patterns in trading activity, Page: 15, line: 468–470.**

"We also observed similar power-law patterns in the out-of-sample validation for Period 4, supporting the robustness of the results across independent time windows."

**(i) Section: Temporal TL Exponent, Table 5, Page: 19,** **Column: 2-7, Row:4, 8, 12, 16, 20, 24, 28, and 32.**

**(j) Section: Temporal TL Exponent, Page: 18, line: 516–518.**

"Similar Taylor's law patterns are observed in the out-of-sample validation (Period 4), supporting the robustness of these results across independent time windows."

**Reviewer’s Comment: Clarify that EOA<>human and SC<>algorithm by default (bots operate via EOAs and many SCs proxy off‑chain human intent); treat this mapping as interpretive and add sensitivity checks (exclude airdrops/exchange flows; stratify major contract types).**

**Authors' Response:** We thank the reviewer for the valuable comment and important clarification.

We agree with the reviewer's observation. In the original manuscript, the terms human-driven and algorithm-driven were used as general descriptive conventions to distinguish EOA and SC transaction types, following terminology adopted in the blockchain research literature [1]. We acknowledge that this framing is an oversimplification, as EOAs may include automated bots and some SCs proxy off-chain human intent.

In the revised manuscript, we have replaced human-driven with EOA-driven and algorithm-driven with SC-driven. The classification now strictly reflects the Ethereum protocol-level distinction between account types rather than implying behavioral intent.

Changes were made at the following locations:

- **Section: Abstract, Page: 1, line: 38.**
- **Section: Introduction, Page: 2, line: 74-75, and 89.**
- **Section: Introduction, Page: 3, line: 108, and 122.**
- **Section: ERC20 Transaction Dataset, Page: 4, line: 172.**
- **Section: Power Law Distribution, Page: 6, line: 234.**
- **Section: Conclusion, Page: 23, line: 600-601.**

***Earlier version: “***One of the unique features of Ethereum is its dual-account model, consisting of Externally Owned Accounts (EOAs), which represent individual users.”

***Revised version: “***One of the unique features of Ethereum is its dual-account model, consisting of Externally Owned Accounts (EOAs), which are controlled by private keys.”

Please see, **Section: *Introduction*, Page: 2, line: 67–68.**

Regarding the sensitivity checks, excluding airdrop and exchange flows and stratifying transactions by contract type would indeed strengthen the analysis. However, performing these checks requires labeled address datasets (e.g., Etherscan tags), which are not available in our current dataset. We plan to incorporate such sensitivity analyses in future work when appropriately labeled data become available.

In the main text we have mentioned in **Conclusion, Page: 23 line: 608–613**:

"Subsequent studies will incorporate sensitivity analyses that exclude known airdrop and exchange flows and stratify smart contract interactions by contract type, and extend the analysis to include graph topological features such as clustering coefficient, modularity, and node centrality to provide a more complete structural characterisation of ERC20 transaction networks."

**Reviewer’s Comment: Remove or substantiate the “****crypto crash” explanation in Period 3 and align the citation to the actual timeframe analyzed.**

**Authors' Response:** Our sincere thanks to the reviewer for the valuable suggestion. We have removed “crypto crash’’ in the revised manuscript.

**Reviewer’s Comment: Reconcile internal inconsistencies (e.g., SC–SC α is 0.67 in Table 2 but 0.62 in the text for Period 3) and ensure text, tables, and figures tell the same story.**

**Authors' Response:** We would like to thank the reviewer for pointing out these inconsistencies.

We have corrected the SC–SC α value from 0.62 to 0.67. We have incorporated the change in the revised manuscript, please see, **Section: Scaling Relationship between Trade Volume and Partner Diversity, Page: 9, line: 385.**

**Reviewer’s Comment : Add light‑weight uncertainty and robustness reporting (brief CIs, sample sizes used in fits; summarize key sensitivity checks) without expanding methods excessively.**

**Authors' Response:** We thank the reviewer for this valuable suggestion.

In the revised manuscript, we have added the light‑weight uncertainty and robustness reporting: 95% confidence intervals (CI) and sample sizes (N) to all results tables. Please see below the list of the revisions:

| Table | Section | Location (Page / Columns) | Added in revised text | Details |
| --- | --- | --- | --- | --- |
| Table 2 | Scaling Relationship between Trade Volume and Partner Diversity | Page 10, Columns 4–6 | 95% CI, R², N | Added for all scaling exponents α across all transaction types and periods. |
| Table 3 | Power-law Patterns in Trading Activity | Page 14, Column 5 | 95% CI | Added for all power-law exponents γ across all transaction types, periods, and sender/receiver roles.  *Note: “95% confidence intervals” also added to the caption of Table 3.* |
| Table 5 | Temporal TL Exponent | Page 19, Columns 5–7 | 95% CI, R², N | Added for all Taylor’s Law exponents β across all transaction types, periods, and sender/receiver roles. |

**Reviewer’s Comment: Improve figure readability: avoid overreliance on log-binned PDFs, label axes consistently, and annotate fitted ranges and thresholds directly on plots.**

**Authors' Response:** Following the reviewer’s suggestions, accordingly we have now revised all figures to improve their readability in the present text. The changes are listed below:

1. Annotating fitted ranges and thresholds on plots.

**Section: Power-law patterns in trading activity, Figures 4–7, Pages 15–18** — all the power-law figures are updated in the revised version. The lower bound of the power-law fitting region, xmin, is now reported directly in the legend of each subplot. A vertical red dashed line has also been added on each plot to indicate the upper threshold, derived by Kolmogorov-Smirnov distance, where the empirical data begins to deviate from the power-law fit in Figs. 4,5,6, and 7. Below, we have shown one of the revised figures.

2. Improve figure readability and consistent axis labeling.

- **Section: Scaling Relationship, Figures 1–3, Pages 11–13** — Axis tick marks standardised to three major ticks; 95% CI and R² added directly on each plot.
- **Section: Power-law Patterns, Figures 4–7, Pages 15–18** — Axis tick marks standardised; xmin reported in legend; vertical red dashed threshold line added. A uniform axis range was not applied as varying dynamic ranges differ across transaction types and time periods.
- **Section: Temporal Taylor's Law, Figures 8–11, Pages 20–23** — Axis tick marks standardised; 95% CI and R² added directly to each plot.

3. Log-binned PDFs.

Log-binned PDFs have been retained as they are a standard approach for visualising heavy-tailed distributions. All scaling exponents are estimated using Maximum Likelihood Estimation (MLE) following Clauset et al. (2009), which is entirely independent of the binning choice used for visualisation.

**Reviewer’s Comment: Make reproducibility concrete: provide code, exact data snapshot/version, and a short README that regenerates Tables/Figures end‑to‑end; specify key preprocessing choices.**

**Authors' Response:** We thank the reviewer for this important suggestion on reproducibility. In the revised manuscript, we have included Code and Data Availability Statements, providing the GitHub repository and detailed instructions to ensure full reproducibility of the analysis.

For revisions in the main text, please see **Section: Code Availability, Pages 24, Line 631-634.**

**Code Availability**

All the source codes for scaling law, power law, Taylor's law, and gas fee analysis are available at:

https://github.com/KundanMukhiaResearchScholar1997/Analysis-of-EOAs-and-Smart-Contracts-in-ERC20-Token-Netwrok.

**Regarding the data availability:**

We sincerely regret that we are unable to upload the raw data directly due to its large size (approximately 50–60 GB). The data snapshots used in this study are specified in main text, please see, **Section: Data Availability, Pages 24, Line 635-639.** Below is the snapshot:

**Data Availability**

The minimal processed dataset required to replicate all statistical analyses in this paper is publicly available at figshare: <https://doi.org/10.6084/m9.figshare.31959735>. The raw Ethereum blockchain transaction data used in this study are publicly available from the XBlock-ETH dataset for the block ranges listed in Table 7.

**Reviewer’s Comment : Define the unit of analysis cleanly (per‑token vs. pooled across tokens) and state whether diversity/volume are token‑specific or aggregated.**

**Authors' Response:** We thank the reviewer for this valuable comment and clarification.

In this study, the unit of analysis is the individual Ethereum address, whether it is an Externally Owned Account (EOA) or a Smart Contract (SC). Our analysis is conducted in a pooled across-tokens framework rather than on a per-token basis. For each address, we combine all ERC-20 transfer events within the study period, rather than analysing tokens separately. Trading volume is defined as the total number of transfer transactions an address sends or receives, regardless of token type or amount. Partner diversity is defined as the number of distinct counterparties an address interacts with. Thus, both measures are aggregated across tokens and are not token-specific.

Changes incorporated in the revised manuscript:

**(a) Section: ERC20 Transaction Dataset, Page: 4, line: 164–167.**

"In this study, the analysis is conducted at the level of individual Ethereum addresses using a pooled framework across tokens rather than a per-token approach. All ERC-20 transfer events associated with an address within a given period are combined before analysis."

**(b) Section: Scaling Law, Page: 5, line: 201–202.**

‘’Both Vᵢ and Nᵢ are calculated by aggregating transactions across all ERC-20 tokens within the given period.’’

# Response to the comments of the Reviewer 3.

**Reviewer’s Comment: The dataset spans from July 2017 to March 2018. However, the Ethereum ecosystem has undergone fundamental paradigm shifts since 2018.**

**Authors' Response:** We thank the reviewer for this important suggestion.

To address the significant evolution of the Ethereum ecosystem since 2018, we have conducted out-of-sample validation using ERC20 transaction data from December 2019 to February 2020. The total number of transactions in this period is 36,692,530. We applied identical methodology, scaling law, power-law distribution, and temporal Taylor's law analysis to this validation dataset. We consider this validation period as Period 4 in our study. The results of Period 4 are consistent with Periods 1–3, confirming that the qualitative distinction between EOA and SC behavioural signatures is robust across two independent time periods.

The following plots illustrate the results from scaling law analysis on Period 4 for EOA-EOA, EOA-SC, SC-EOA, and SC-SC transactions. We have incorporated the scaling law plot for Period 4 in the supporting information. Please see, **Supporting Information Fig. S1.**

In the revised manuscript, we have incorporated the following changes:

Please refer to the detailed revisions provided to address the similar comments made by Reviewer 2, which also address this present concern. The specific revisions have been incorporated in the following locations in the manuscript:

**(a) Section: Abstract, Page: 1, line: 19–22.**

**(b) Section: ERC20 Transaction Dataset, Page: 3-4, line: 139–145.**

**(c) Section: ERC20 Transaction Dataset, Page: 4, line: 162–167.**

**(d) Section: ERC20 Transaction Dataset, Table 1, Page: 4, columns 7 and 8.**

**(e) Section: Scaling Relationship between Trade Volume and Partner Diversity, Table 2, Page: 10, Column: 2-6 and 3, Row: 4, 8, 12, and 16.**

**(f) Section: Scaling Relationship between Trade Volume and Partner Diversity, Page: 9, line: 386–390. (g)Section: Power-law patterns in trading activity, Table 3, Page: 14, Column: 2-10, Row:7, 8, 15, 16, 23, 24, 31, and 32.**

**(h) Section: Power-law patterns in trading activity, Page: 15, line: 468–470.**

**(i) Section: Temporal TL Exponent, Table 5, Page: 19, Column: 2-7, Row:4, 8, 12, 16, 20, 24, 28, and 32.**

**(j) Section: Temporal TL Exponent, Page: 18, line: 516–518.**

**Reviewer’s Comment: The paper inaccurately assumes that all Externally Owned Accounts (EOAs) represent "human-driven" behavior, ignoring the fact that many EOAs are actually automated high-frequency trading bots.**

**Authors' Response:** We thank the reviewer for the valuable comment and important clarification.

We agree with the reviewer's observation. In the original manuscript, the terms human-driven and algorithm-driven were used as general descriptive conventions to distinguish EOA and SC transaction types, following terminology adopted in the blockchain research literature [1]. We acknowledge that this framing is an oversimplification, as EOAs may include automated bots and some SCs proxy off-chain human intent.

In the revised manuscript, we have replaced human-driven with EOA-driven and algorithm-driven with SC-driven throughout. The classification now strictly reflects the Ethereum protocol-level distinction between account types rather than implying behavioural intent. Changes were applied at the following locations:

- **Section: Abstract, Page: 1, line: 38.**
- **Section: Introduction, Page: 2, line: 74-75, and 89.**
- **Section: Introduction, Page: 3, line: 108, and 122.**
- **Section: ERC20 Transaction Dataset, Page: 4, line: 172.**
- **Section: Power Law Distribution, Page: 6, line: 234.**
- **Section: Conclusion, Page: 23, line: 600-601.**

***Earlier version:*** *“*One of the unique features of Ethereum is its dual-account model, consisting of Externally Owned Accounts (EOAs), which represent individual users.”

***Revised version:*** *“*One of the unique features of Ethereum is its dual-account model, consisting of Externally Owned Accounts (EOAs), which are controlled by private keys.”

Please see, **Section: Introduction, Page: 2, line: 67–68.**

**Reviewer’s Comment: The arbitrary selection of a 1-hour time window to compute the mean and variance for Temporal Taylor's Law lacks validation, as scaling laws are often highly sensitive to time-scale dependencies.**

**Authors' Response:** We thank the reviewer for raising the point.

To test the robustness of our results with respect to the choice of time scale, we conducted a sensitivity analysis using three different time windows: 30 minutes, 1 hour, and 2 hours. The detailed results for the 30-minute and 2-hour windows are presented in Supporting Information S1 Table and S2 Table, respectively. Our analysis shows that Taylor's Law exponent β remains consistent across these different time windows, indicating that the results are not sensitive to the choice of time window in our dataset.

In the revised manuscript, we have incorporated the following changes:

**(a) Section: Temporal TL Exponent, Page: 19, line: 538–542.**

"To further validate the robustness of the TL exponents with respect to time-scale selection, we conducted a sensitivity analysis using 30-minute and 2-hour aggregation windows. The S1 Table and S2 Table show that the exponent β remains stable across all three time windows for all transaction types and periods, within the reported confidence intervals (CI)."

**(b) Section: Supporting Information, Page: 23, line: 616–630.**

S1 Table caption: "TL exponents β for 30-minute time window. Sensitivity analysis of Taylor's Law exponents across all transaction types and periods using a 30-minute aggregation window."

S2 Table caption: "TL exponents β for 2-hour time window. Sensitivity analysis of Taylor's Law exponents across all transaction types and periods using a 2-hour aggregation window.

**Reviewer’s Comment: Despite framing the research within complex systems theory, the analysis focuses purely on degree and volume distributions, ignoring critical graph topological features like clustering coefficient, modularity, or node centrality.**

**Authors' Response:** We sincerely thank the reviewer for this insightful suggestion.

We agree that graph topological measures such as clustering coefficient, modularity, and node centrality can provide valuable complementary insights into the structural organisation of transaction networks. However, the primary objective of this study is to investigate the statistical behavioural properties of ERC20 trading activity at the individual account level, focusing on scaling laws, power-law distributions, and Temporal Taylor's Law. Incorporating a detailed graph-topological analysis would require constructing and analysing the full transaction network, which lies beyond the intended scope of the present work. We appreciate the reviewer's suggestion and have acknowledged it in the revised manuscript as a promising direction for future research.

In the revised manuscript, we have incorporated the following changes in **Section: Conclusion,** **Page: 23, line: 608–613**:

"Subsequent studies will incorporate sensitivity analyses that exclude known airdrop and exchange flows and stratify smart contract interactions by contract type, and extend the analysis to include graph topological features such as clustering coefficient, modularity, and node centrality to provide a more complete structural characterisation of ERC20 transaction networks."

**Reviewer’s Comment: The paper's novelty is limited as it merely applies off-the-shelf complex network tools to blockchain data, yielding highly intuitive and purely descriptive conclusions without deeper theoretical insights. To enhance originality, the authors should move beyond mere statistical observation by incorporating blockchain-specific constraints (such as gas fees or contract composability) and proposing models to explain the underlying mechanisms driving these distinct behavior.**

**Authors' Response:** We thank the reviewer for this constructive feedback.

We respectfully clarify that the contribution of this study lies in the systematic empirical characterisation of EOA-driven and SC-driven transactions using scaling laws, power-law distributions, and Temporal Taylor's Law across a large-scale dataset spanning over 81 million ERC20 transactions across two independent periods.

As per the reviewer's valuable suggestion, we have strengthened the manuscript by incorporating a blockchain-specific gas fee analysis. Using 210.1 million raw Ethereum transactions across four periods, we show that EOA–SC transactions consistently incur higher costs than EOA–EOA transactions, with a mean ratio of approximately 3.04 across periods. This provides direct empirical evidence of the higher computational complexity associated with smart contract execution and complements the statistical findings with a blockchain-specific mechanism.

We have revised the manuscript. In the revised manuscript, we added a two new section, and one table:

(a) **Block Transaction Dataset:** Please see **Section: Block Transaction Dataset, Page 5, lines 183–194.**

‘’To analyze transaction gas fees on the Ethereum blockchain, we used block transaction data corresponding to the same four time periods defined in Section (ERC20 Transaction Dataset). The analysis spans Period 1 (July–September 2017), Period 2 (October–December 2017), and Period 3 (January–March 2018), which together constitute the primary dataset, while Period 4 (December 2019–February 2020) serves as an out-of-sample validation period. The dataset includes transaction-level information such as gasUsed and gasPrice, which are used to compute the gas fee associated with each transaction. The gas fee (in Gwei) is calculated as[2]:

Gas Fee = (gasUsed × gasPrice) / 10^9^

Here, gasPrice is originally expressed in Wei, the smallest unit of Ether, where 1 Gwei = 10^9^ Wei. Therefore, division by 10^9^ converts the gas fee into Gwei.’’

(b) **Gas Fee Analysis:** Please see **Pages: 20–21, lines: 544–574, and Table 6.**

**Gas Fee Analysis**

Gas fees reflect the computational cost of executing different transaction types on Ethereum. We analyze gas fee statistics using raw Ethereum block transaction data comprising 210,052,577 EOA-initiated transactions across all four periods. Gas fees are computed as the product of gasUsed and gasPrice. We convert this value to Gwei by dividing by 10⁹, where 1 ETH equals 10⁹ Gwei, ensuring a consistent unit of measurement across all transactions. Table X presents descriptive statistics of gas fees for EOA-initiated transactions across all four periods. Since Ethereum transactions must be initiated by EOA, SC cannot independently initiate transactions. SC–EOA and SC–SC interactions represent internal message calls that are not recorded as independent transactions in the block transaction data, and are therefore not present in the gas fee analysis. The gas cost of internal calls SC–EOA and SC–SC cannot be measured separately at the block level. They are included in the total gas used by the main EOA–SC transaction. Therefore, the gas fees shown for EOA–SC transactions in Table 6 represent the cost of the entire sequence of internal calls, not just the initial interaction between the user and the contract. EOA–SC transactions consistently exhibit substantially higher mean gas fees compared to EOA–EOA transactions, with a mean ratio of approximately 3.04 across all periods (Table Y). This difference directly reflects the greater computational complexity of smart contract execution relative to simple EOA–EOA transfers. The lower absolute gas fee values observed in Period 4, with EOA–EOA at 247,509 Gwei and EOA–SC at 813,664 Gwei, compared to Periods 1–3, are consistent with the generally lower gas prices prevailing during 2019–2020. Importantly, despite these differences in absolute values, the ratio between EOA–SC and EOA–EOA gas fees remains stable across all periods, ranging from 2.31 to 3.44, confirming that the structural cost difference between transaction types is persistent and independent of market conditions. The large standard deviation relative to the mean in both transaction types indicates a highly skewed distribution of gas fees, consistent with the heavy-tailed trading activity distributions reported in the power-law analysis. These blockchain-specific observations provide additional empirical support for the structural and behavioral distinctions between EOA-driven and SC-driven interactions reported throughout this study.”

(c) Please see, **Section: Conclusion, Pages: 22-23, lines: 595-599.**

‘’A gas fee analysis further confirms this distinction, showing that EOA--SC transactions consistently incur approximately 3 times higher gas fees than EOA--EOA transactions across all periods, providing blockchain-specific empirical evidence for the greater computational complexity of smart contract execution.’’

(d) Please see, **Section: Abstract, Pages: 1, lines: 34-37.**

‘’The gas fee analysis further confirms the difference between EOA and SC, with EOA-SC transactions incurring much higher gas fees, reflecting the greater computational complexity and reinforcing the structural differences between EOA-driven activity and smart contract involvement.’’

# References

[1] Chen, W., Zheng, Z., Ngai, E.C.H., Zheng, P. and Zhou, Y., Traveling the token world: A graph analysis of Ethereum ERC20 token ecosystem. Proceedings of the Web Conference 2020, 1411–1421 (2020).

# [2] Liu, Y., Lu, Y., Nayak, K., Zhang, F., Zhang, L. and Zhao, Y., Empirical analysis of EIP-1559: Transaction fees, waiting times, and consensus security. Proceedings of the 2022 ACM SIGSAC Conference on Computer and Communications Security, 2099–2113 (2022)
