# Supplementary material for: Statistical patterns in the Ethereum blockchain: Analysis of EOAs and smart contracts in ERC20 token network
Source: PLoS One. 2026 Oct 1;21(10):e0358972. doi: 10.1371/journal.pone.0358972 (PMC13630221; doi:10.1371/journal.pone.0358972)
Supplement: Supplementary file 5 [file pone.0358972.s006.docx]

# Response to Reviewer Comments

We extend our sincere thanks to the editors and reviewers for the thoughtful suggestions and comments, which helped to improve the quality and clarity of our manuscript. We have carefully addressed each of the reviewer comments point by point, and all corresponding revisions have been incorporated into the revised manuscript. Detailed responses to each comment are provided below, with changes in the manuscript appropriately highlighted.

# Summary of the revisions

# In the revised manuscript, we have highlighted our revisions in red-colored text. Below is a brief summary of the revisions made in response to Reviewer comments.

# 1. Removal of Algorithm-Driven Language: We have revised the abstract and other sections to avoid implying EOA = human-driven or SC = algorithm-driven by default. The abstract now uses "protocol-level activity" in place of "algorithm-driven activity.

# 2. Terminological consistency: We have corrected all occurrences of "individual tokens" to "individual accounts" throughout the manuscript, consistent with the methods section, which states that the analysis is conducted at the level of individual Ethereum addresses..

# 3. Gas-fee interpretation: We have softened the interpretation of gas-fee results throughout the manuscript. The phrases "directly reflects" and "confirms" have been replaced with "is consistent with" and "supports." We have also added a sentence explaining that gas fees depend on both gasUsed and gasPrice, and therefore do not represent computational cost alone. These changes have been made in the Abstract, Gas fee analysis section, and Conclusion sections.

# 4. Universal claims: We have qualified all claims of universality with more appropriate phrasing.

# 5. GitHub URL Correction: We have corrected the typographical error "Netwrok" to "Network" in the Code Availability section. We have also verified that the GitHub repository is publicly accessible at: <https://github.com/KundanMukhiaResearchScholar1997/Analysis-of-EOAs-and-Smart-Contracts-in-ERC20-Token-Network>

# REVIEWER COMMENTS:

**Reviewer’s Comment: Please remove or qualify any remaining language that could imply EOA = human-driven or SC = algorithm-driven by default. Although the response letter states that such wording has been revised, the abstract still states that SC-driven activity indicates “algorithm-driven activity.” This should be changed to a more protocol-level description, such as “contract-mediated,” “protocol-level,” or simply “SC-driven” activity.**

**Authors' Response:** We sincerely thank the reviewer for this helpful suggestion. We have carefully revised the manuscript to remove wording that could imply that EOAs are human-driven or that SCs are algorithm-driven. We have replaced the phrase "algorithm-driven activity" with "protocol-level activity" in the Abstract. Similar wording has also been checked throughout the manuscript to ensure consistent terminology. The relevant changes incorporated into the revised manuscript are provided below.

**Original**: Moreover, SC-driven activity displays heavier-tailed distributions (γ < 2), indicating bursty and algorithm-driven activity.

**Revised**: [Section: Abstract, Page: 1, Line: 34]: Moreover, SC-driven activity displays heavier-tailed distributions (γ < 2), indicating bursty and protocol-level activity.

**Original:** Beyond categorizing transactional roles, this study emphasizes how decentralized finance (DeFi) ecosystems are shaped by the interplay of EOA-driven decisions and algorithmic executions.

**Revised**: [Section: Introduction, Page: 3, Line: 126]: Beyond categorizing transactional roles, this study emphasizes how decentralized finance (DeFi) ecosystems are shaped by the interplay of EOA-driven decisions and SC-driven executions.

**Reviewer’s Comment: Please ensure terminological consistency regarding the unit of analysis. The Methods state that the analysis is conducted at the level of individual Ethereum addresses and pooled across tokens, rather than as a per-token analysis. However, the Temporal Taylor’s Law section refers to “individual tokens.” This should be revised to “individual accounts” or “individual addresses.” Similar token/account wording should also be checked in the power-law section.**

**Authors' Response**: We agree, and thank the reviewer for pointing this out. We carefully reviewed the manuscript and revised the terminology by replacing "individual tokens" with "individual accounts," consistent with the methodology adopted in this study. We also checked the Power-law and Temporal Taylor's Law sections to ensure consistent terminology throughout the manuscript. The relevant changes incorporated into the revised manuscript are provided below.

**Original:** To understand the trading behavior in ERC20 token transactions, we study whether the number of trades made or received by tokens follows a power-law distribution.

**Revised**: [Section: Power-law patterns in trading activity, Page: 10, Line: 395]: To understand the trading behavior in ERC20 token transactions, we study whether the number of trades made or received by traders follows a power-law distribution.

**Original**: The lower values of γ indicate the more extreme concentration, i.e., a few tokens dominate the trading activity.

**Revised:** [Section: Power-law patterns in trading activity, Page: 10, Line: 402]: The lower values of γ indicate the more extreme concentration, i.e., a few accounts dominate the trading activity.

**Original**: It captures the relationship between the temporal mean and variance of token trades for individual tokens.

**Revised**: [Section: Temporal TL Exponent, Page: 16, Line: 492]: It captures the relationship between the temporal mean and variance of token trades for individual accounts.

**Reviewer’s Comment: Please qualify the gas-fee interpretation. Since gas fee is computed as gasUsed × gasPrice, higher mean gas fees are consistent with greater computational complexity of smart contract execution, but do not by themselves isolate computational cost from gas-price or fee-market effects. The authors could either soften the wording from “directly reflects” to “is consistent with,” .**

**Authors' Response:** We sincerely thank the reviewer for this important observation. We have revised the manuscript to avoid overstating the interpretation of gas fees. Statements such as "directly reflects" and "confirms" have been replaced with more appropriate wording, including "is consistent with" and "supports." We have also added a clarification that gas fees depend on both gasUsed and gasPrice and therefore do not represent computational cost alone. The relevant changes incorporated into the revised manuscript are provided below.

## Original: This difference directly reflects the greater computational complexity of smart contract execution relative to simple EOA--EOA transfers.

**Revised:** [Section: Gas Fee Analysis, Page: 20, Line: 562-565]: This difference is consistent with the greater computational complexity of smart contract execution relative to simple EOA–EOA transfers. The gas fee, as the product of gasUsed and gasPrice, cannot by itself isolate computational cost from gas-price and fee-market effects.

## Original: Importantly, despite these differences in absolute values, the ratio between EOA--SC and EOA--EOA gas fees remains stable across all periods, ranging from 2.31 to 3.44, confirming that the structural cost difference between transaction types is persistent and independent of market conditions.

**Revised:** [Section: Gas Fee Analysis, Page: 21, Line: 568-572]: Importantly, despite these differences in absolute values, the ratio between EOA--SC and EOA--EOA gas fees remains stable across all periods, ranging from 2.31 to 3.44, suggesting that the structural cost difference persists across periods, though the ratio reflects both computational and market factors.

## Original: A gas fee analysis further confirms this distinction, showing that EOA--SC transactions consistently incur approximately 3 times higher gas fees than EOA--EOA transactions across all periods, providing blockchain-specific empirical evidence for the greater computational complexity of smart contract execution.

## Revised: [Section: Conclusion, Page: 22-23, Line: 598-602]: A gas fee analysis further support this distinction, showing that EOA--SC transactions consistently incur approximately 3 times higher gas fees than EOA--EOA transactions across all periods, consistent with the greater computational complexity of smart contract execution, while also reflecting gas-price and fee-market dynamics.

**Original:** The gas fee analysis further confirms the difference between EOA and SC, with EOA-SC transactions incurring much higher gas fees, reflecting the greater computational complexity and reinforcing the structural differences between EOA-driven activity and SC involvement.

**Revised:** [Section:Abstract, Page: 1, Line: 34-37]: The gas fee analysis further supports the difference between EOA and SC, with EOA-SC transactions incurring much higher gas fees, consistent with the greater computational complexity of smart contract execution and reinforcing the structural differences between EOA-driven activity and SC involvement.

**Reviewer’s Comment : Please moderate remaining claims about “universal” behavior. The results support robust empirical regularities in the analyzed ERC20 data, especially the contrast between EOA-driven and SC-driven interaction patterns. However, broader universality across chains, contract types, and behavioral regimes remains a future-work question. Phrases such as “universal feature,” “near-universal statistical patterns,” or “not governed by universal dynamics” should be qualified accordingly.**

**Authors' Response:** We thank the reviewer for this valuable suggestion. We have carefully revised the manuscript to qualify statements related to universality. Terms such as "universal feature," "near-universal statistical patterns," and "not governed by universal dynamics" have been modified to better reflect that our findings are based on the analyzed ERC20 dataset. The relevant changes incorporated into the revised manuscript are provided below.

## Original: However, the presence of heavy-tailed distributions is a universal feature, reflecting the unequal and scale-free nature of transactional activity in the ERC20 ecosystem.

## Revised: [Section: Power-law patterns in trading activity, Page: 13, Line: 451-453]: However, the presence of heavy-tailed distributions is a consistent statistical pattern across all transaction types in our dataset, reflecting the unequal and scale-free nature of transactional activity in the ERC20 ecosystem.

## Original: Both analyses indicate that EOA-based transactions exhibit more universal and scalable behavior, while SC-based activities, particularly SC-SC, appear more context-dependent and structurally variable.

## Revised: [Section: Power-law patterns in trading activity, Page: 14, Line: 463-465]: Both analyses indicate that EOA-based transactions exhibit more consistent and scalable behavior, while SC-based activities, particularly SC-SC, appear more context-dependent and structurally variable.

## Original: We employed temporal TL to further evaluate whether the trading behavior observed in ERC20 token interactions reflects universal patterns.

## Revised: [Section: Temporal TL Exponent, Page: 16, Line: 486-487]: We employed temporal TL to further evaluate whether the trading behavior observed in ERC20 token interactions reflects statistical patterns.

## Original: for receivers. This indicates that interactions driven by EOA tend to follow consistent and near-universal statistical patterns.

## Revised: [Section: Temporal TL Exponent, Page: 18, Line: 530-531]: This indicates that interactions driven by EOA tend to follow consistent and statistical patterns.

## Original: for receivers. These findings indicate that EOA-driven blockchain transactions follow universal patterns observed in traditional economic and social systems.

## Revised: [Section: Conclusion, Page: 21, Line: 586-587]: These findings indicate that EOA-driven blockchain transactions follow statistical patterns observed in traditional economic and social systems.

**Reviewer’s Comment : Please verify the GitHub repository URL in the Code Availability section. The repository path contains “Netwrok,” which appears to be a typographical error for “Network” unless this is the actual repository name. Since the link is central to reproducibility, the final manuscript should ensure that the URL is correct and accessible.**

**Authors' Response:** We thank the reviewer for pointing out this typographical error. We have corrected the repository name from "Netwrok" to "Network" in the Code Availability section. We also verified that the repository is publicly accessible. The relevant changes incorporated into the revised manuscript are provided below.

**Original:** <https://github.com/KundanMukhiaResearchScholar1997/Analysis-of-EOAs-and-Smart-Contracts-in-ERC20-Token-Netwrok>

**Revised:** [Section: Code Availability, Page: 24, Line: 637-638]:

<https://github.com/KundanMukhiaResearchScholar1997/Analysis-of-EOAs-and-Smart-Contracts-in-ERC20-Token-Network>
