# Supplementary material for: Statistical patterns in the Ethereum blockchain: Analysis of EOAs and smart contracts in ERC20 token network
Source: PLoS One. 2026 Oct 1;21(10):e0358972. doi: 10.1371/journal.pone.0358972 (PMC13630221; doi:10.1371/journal.pone.0358972)
Supplement: Peer review history

# Peer review: Decision Letter 0

**DOI:** 10.1371/journal.pone.0358972.r001

**Authors:** Junhuan Zhang

27 Feb 2026

Dear Dr. Saha,

Thank you for submitting your manuscript to PLOS ONE. After careful consideration, we feel that it has merit but does not fully meet PLOS ONE’s publication criteria as it currently stands. Therefore, we invite you to submit a revised version of the manuscript that addresses the points raised during the review process.

Please submit your revised manuscript by Apr 13 2026 11:59PM. If you will need more time than this to complete your revisions, please reply to this message or contact the journal office at plosone@plos.org. When you're ready to submit your revision, log on to https://www.editorialmanager.com/pone/ and select the 'Submissions Needing Revision' folder to locate your manuscript file.

- A letter that responds to each point raised by the academic editor and reviewer(s). You should upload this letter as a separate file labeled 'Response to Reviewers'.
- A marked-up copy of your manuscript that highlights changes made to the original version. You should upload this as a separate file labeled 'Revised Manuscript with Track Changes'.
- An unmarked version of your revised paper without tracked changes. You should upload this as a separate file labeled 'Manuscript'.

If you would like to make changes to your financial disclosure, please include your updated statement in your cover letter. Guidelines for resubmitting your figure files are available below the reviewer comments at the end of this letter.

If applicable, we recommend that you deposit your laboratory protocols in protocols.io to enhance the reproducibility of your results. Protocols.io assigns your protocol its own identifier (DOI) so that it can be cited independently in the future. For instructions see: https://journals.plos.org/plosone/s/submission-guidelines#loc-laboratory-protocols. Additionally, PLOS ONE offers an option for publishing peer-reviewed Lab Protocol articles, which describe protocols hosted on protocols.io. Read more information on sharing protocols at https://plos.org/protocols?utm_medium=editorial-email&utm_source=authorletters&utm_campaign=protocols.

We look forward to receiving your revised manuscript.

Kind regards,

Junhuan Zhang, PhD

Academic Editor

PLOS One

Journal Requirements:

When submitting your revision, we need you to address these additional requirements.

1. Please ensure that your manuscript meets PLOS ONE's style requirements, including those for file naming. The PLOS ONE style templates can be found at

https://journals.plos.org/plosone/s/file?id=wjVg/PLOSOne_formatting_sample_main_body.pdf and

https://journals.plos.org/plosone/s/file?id=ba62/PLOSOne_formatting_sample_title_authors_affiliations.pdf

2. Thank you for stating the following in the Acknowledgments Section of your manuscript:

“The authors acknowledge the National Institute of Technology Sikkim for allocating doctoral research fellowships to Kundan Mukhia, and S.R. Luwang.”

We note that you have provided funding information that is not currently declared in your Funding Statement. However, funding information should not appear in the Acknowledgments section or other areas of your manuscript. We will only publish funding information present in the Funding Statement section of the online submission form.

Please remove any funding-related text from the manuscript and let us know how you would like to update your Funding Statement. Currently, your Funding Statement reads as follows:

“Doctoral research fellowships to Kundan Mukhia, and S.R. Luwang are funded by the National Institute of Technology Sikkim India”

Please include your amended statements within your cover letter; we will change the online submission form on your behalf.

3. Thank you for stating the following financial disclosure:

“Doctoral research fellowships to Kundan Mukhia, and S.R. Luwang are funded by the National Institute of Technology Sikkim India”

Please state what role the funders took in the study.  If the funders had no role, please state: "The funders had no role in study design, data collection and analysis, decision to publish, or preparation of the manuscript."

If this statement is not correct you must amend it as needed.

Please include this amended Role of Funder statement in your cover letter; we will change the online submission form on your behalf.

4. Please provide a complete Data Availability Statement in the submission form, ensuring you include all necessary access information or a reason for why you are unable to make your data freely accessible. If your research concerns only data provided within your submission, please write "All data are in the manuscript and/or supporting information files" as your Data Availability Statement.

5. Please include your full ethics statement in the ‘Methods’ section of your manuscript file. In your statement, please include the full name of the IRB or ethics committee who approved or waived your study, as well as whether or not you obtained informed written or verbal consent. If consent was waived for your study, please include this information in your statement as well.

6. If the reviewer comments include a recommendation to cite specific previously published works, please review and evaluate these publications to determine whether they are relevant and should be cited. There is no requirement to cite these works unless the editor has indicated otherwise.

[Note: HTML markup is below. Please do not edit.]

Reviewers' comments:

Reviewer's Responses to Questions

**Comments to the Author**

1. Is the manuscript technically sound, and do the data support the conclusions?

Reviewer #2: Partly

Reviewer #3: Yes

**********

2. Has the statistical analysis been performed appropriately and rigorously?

Reviewer #2: Yes

Reviewer #3: Yes

**********

3. Have the authors made all data underlying the findings in their manuscript fully available?

The PLOS Data policy

Reviewer #2: No

Reviewer #3: Yes

**********

4. Is the manuscript presented in an intelligible fashion and written in standard English?

Reviewer #2: Yes

Reviewer #3: Yes

**********

Reviewer #2: The framing is promising and the visuals communicate well, but the claim of “universal” patterns seem to stretch beyond what a single, atypical period and broad aggregations can safely support.

Advice to strengthen the manuscript

Hedge “universal” language; anchor claims to the July 2017–March 2018 window stated in the abstract and dataset description, and rephrase as period‑specific until validated. It would be nice to add out‑of‑sample validation (more recent years and, if feasible, another chain) to test stability of the reported regularities before generalizing.

Clarify that EOA<>human and SC<>algorithm by default (bots operate via EOAs and many SCs proxy off‑chain human intent); treat this mapping as interpretive and add sensitivity checks (exclude airdrops/exchange flows; stratify major contract types).

Remove or substantiate the “crypto crash” explanation in Period 3 and align the citation to the actual timeframe analyzed.

Reconcile internal inconsistencies (e.g., SC–SC α is 0.67 in Table 2 but 0.62 in the text for Period 3) and ensure text, tables, and figures tell the same story.

Add light‑weight uncertainty and robustness reporting (brief CIs, sample sizes used in fits; summarize key sensitivity checks) without expanding methods excessively.

Improve figure readability: avoid overreliance on log‑binned PDFs, label axes consistently, and annotate fitted ranges and thresholds directly on plots.

Make reproducibility concrete: provide code, exact data snapshot/version, and a short README that regenerates Tables/Figures end‑to‑end; specify key preprocessing choices.

Define the unit of analysis cleanly (per‑token vs. pooled across tokens) and state whether diversity/volume are token‑specific or aggregated.

Reviewer #3: This manuscript investigates the distinct statistical signatures of human-driven (Externally Owned Accounts, EOAs) and algorithm-driven (Smart Contracts, SCs) transactions on the Ethereum blockchain. By analyzing over 44 million ERC20 token transfers and applying methodologies from complex systems—namely, volume-partner scaling laws, power-law distribution fitting, and temporal Taylor's Law—the authors successfully demonstrate that EOA interactions follow universal, stable behavioral patterns, whereas SC-driven interactions are bursty, highly concentrated, and structurally volatile.

Comments：

1. The dataset spans from July 2017 to March 2018. However, the Ethereum ecosystem has undergone fundamental paradigm shifts since 2018.

2. The paper inaccurately assumes that all Externally Owned Accounts (EOAs) represent "human-driven" behavior, ignoring the fact that many EOAs are actually automated high-frequency trading bots.

3. The arbitrary selection of a 1-hour time window to compute the mean and variance for Temporal Taylor's Law lacks validation, as scaling laws are often highly sensitive to time-scale dependencies.

4. Despite framing the research within complex systems theory, the analysis focuses purely on degree and volume distributions, ignoring critical graph topological features like clustering coefficient, modularity, or node centrality.

5. The paper's novelty is limited as it merely applies off-the-shelf complex network tools to blockchain data, yielding highly intuitive and purely descriptive conclusions without deeper theoretical insights. To enhance originality, the authors should move beyond mere statistical observation by incorporating blockchain-specific constraints (such as gas fees or contract composability) and proposing models to explain the underlying mechanisms driving these distinct behaviors.

**********

what does this mean?). If published, this will include your full peer review and any attached files.

If you choose “no”, your identity will remain anonymous but your review may still be made public.

**Do you want your identity to be public for this peer review?**  For information about this choice, including consent withdrawal, please see our Privacy Policy

Reviewer #2: No

Reviewer #3: No

**********

[NOTE: If reviewer comments were submitted as an attachment file, they will be attached to this email and accessible via the submission site. Please log into your account, locate the manuscript record, and check for the action link "View Attachments". If this link does not appear, there are no attachment files.]

To ensure your figures meet our technical requirements, please review our figure guidelines: https://journals.plos.org/plosone/s/figures

You may also use PLOS’s free figure tool, NAAS, to help you prepare publication quality figures: https://journals.plos.org/plosone/s/figures#loc-tools-for-figure-preparation.

NAAS will assess whether your figures meet our technical requirements by comparing each figure against our figure specifications.

# Author comment: Author response to Decision Letter 1

**DOI:** 10.1371/journal.pone.0358972.r002

8 Apr 2026

We express our sincere thanks to the editor for providing the opportunity to revise and resubmit. We also extend our gratitude to the editor and reviewers for their thoughtful suggestions and comments, which greatly helped to improve our work. We have carefully addressed each of the reviewers' comments point by point. Based on their suggestions, we have made a major overhaul of the main text, with particular attention to rewriting the Abstract, Methods, and Results sections for improved clarity and to better articulate the scientific impact of our findings. In the revised version, we have thoroughly reorganized the Results section, revised the figures, and reproduced the results. In addition, we have undertaken new analyses following the reviewers' recommendations.

Attachment Submitted filename: Response to Reviewers.docx

# Peer review: Decision Letter 1

**DOI:** 10.1371/journal.pone.0358972.r003

**Authors:** Junhuan Zhang

26 Jun 2026

Dear Dr. Saha,

Thank you for submitting your manuscript to PLOS ONE. After careful consideration, we feel that it has merit but does not fully meet PLOS ONE’s publication criteria as it currently stands. Therefore, we invite you to submit a revised version of the manuscript that addresses the points raised during the review process.

Please submit your revised manuscript by Aug 10 2026 11:59PM. If you will need more time than this to complete your revisions, please reply to this message or contact the journal office at plosone@plos.org. When you're ready to submit your revision, log on to https://www.editorialmanager.com/pone/ and select the 'Submissions Needing Revision' folder to locate your manuscript file.

- A letter that responds to each point raised by the academic editor and reviewer(s). You should upload this letter as a separate file labeled 'Response to Reviewers'.
- A marked-up copy of your manuscript that highlights changes made to the original version. You should upload this as a separate file labeled 'Revised Manuscript with Track Changes'.
- An unmarked version of your revised paper without tracked changes. You should upload this as a separate file labeled 'Manuscript'.

If you would like to make changes to your financial disclosure, please include your updated statement in your cover letter. Guidelines for resubmitting your figure files are available below the reviewer comments at the end of this letter.

If applicable, we recommend that you deposit your laboratory protocols in protocols.io to enhance the reproducibility of your results. Protocols.io assigns your protocol its own identifier (DOI) so that it can be cited independently in the future. For instructions see: https://journals.plos.org/plosone/s/submission-guidelines#loc-laboratory-protocols. Additionally, PLOS ONE offers an option for publishing peer-reviewed Lab Protocol articles, which describe protocols hosted on protocols.io. Read more information on sharing protocols at https://plos.org/protocols?utm_medium=editorial-email&utm_source=authorletters&utm_campaign=protocols.

As the corresponding author, your ORCID iD is verified in the submission system and will appear in the published article. PLOS supports the use of ORCID, and we encourage all coauthors to register for an ORCID iD and use it as well. Please encourage your coauthors to verify their ORCID iD within the submission system before final acceptance, as unverified ORCID iDs will not appear in the published article. *Only*  the individual author can complete the verification step; PLOS staff *cannot*  verify ORCID iDs on behalf of authors.

We look forward to receiving your revised manuscript.

Kind regards,

Junhuan Zhang, PhD

Academic Editor

PLOS One

Journal Requirements:

If the reviewer comments include a recommendation to cite specific previously published works, please review and evaluate these publications to determine whether they are relevant and should be cited. There is no requirement to cite these works unless the editor has indicated otherwise.

Please review your reference list to ensure that it is complete and correct. If you have cited papers that have been retracted, please include the rationale for doing so in the manuscript text, or remove these references and replace them with relevant current references. Any changes to the reference list should be mentioned in the rebuttal letter that accompanies your revised manuscript. If you need to cite a retracted article, indicate the article’s retracted status in the References list and also include a citation and full reference for the retraction notice.

[Note: HTML markup is below. Please do not edit.]

Reviewers' comments:

Reviewer's Responses to Questions

**Comments to the Author**

Reviewer #4: (No Response)

**********

2. Is the manuscript technically sound, and do the data support the conclusions?

Reviewer #4: Yes

**********

3. Has the statistical analysis been performed appropriately and rigorously?

Reviewer #4: Yes

**********

4. Have the authors made all data underlying the findings in their manuscript fully available?

The PLOS Data policy

Reviewer #4: Yes

**********

5. Is the manuscript presented in an intelligible fashion and written in standard English?

Reviewer #4: Yes

**********

Reviewer #4: The revised manuscript is substantially improved and, in my view, suitable for publication after minor revisions. The large-scale ERC20 dataset, the distinction between EOA–EOA, EOA–SC, SC–EOA, and SC–SC interactions, the out-of-sample Period 4 validation, and the additional gas-fee analysis make the empirical contribution clearer and more robust. My remaining comments concern clarification, terminology, and over-interpretation rather than the core results.

-Please remove or qualify any remaining language that could imply EOA = human-driven or SC = algorithm-driven by default. Although the response letter states that such wording has been revised, the abstract still states that SC-driven activity indicates “algorithm-driven activity.” This should be changed to a more protocol-level description, such as “contract-mediated,” “protocol-level,” or simply “SC-driven” activity.

-Please ensure terminological consistency regarding the unit of analysis. The Methods state that the analysis is conducted at the level of individual Ethereum addresses and pooled across tokens, rather than as a per-token analysis. However, the Temporal Taylor’s Law section refers to “individual tokens.” This should be revised to “individual accounts” or “individual addresses.” Similar token/account wording should also be checked in the power-law section.

-Please qualify the gas-fee interpretation. Since gas fee is computed as gasUsed × gasPrice, higher mean gas fees are consistent with greater computational complexity of smart contract execution, but do not by themselves isolate computational cost from gas-price or fee-market effects. The authors could either soften the wording from “directly reflects” to “is consistent with,” .

-Please moderate remaining claims about “universal” behavior. The results support robust empirical regularities in the analyzed ERC20 data, especially the contrast between EOA-driven and SC-driven interaction patterns. However, broader universality across chains, contract types, and behavioral regimes remains a future-work question. Phrases such as “universal feature,” “near-universal statistical patterns,” or “not governed by universal dynamics” should be qualified accordingly.

-Please verify the GitHub repository URL in the Code Availability section. The repository path contains “Netwrok,” which appears to be a typographical error for “Network” unless this is the actual repository name. Since the link is central to reproducibility, the final manuscript should ensure that the URL is correct and accessible.

**********

what does this mean?). If published, this will include your full peer review and any attached files.

If you choose “no”, your identity will remain anonymous but your review may still be made public.

**Do you want your identity to be public for this peer review?**  For information about this choice, including consent withdrawal, please see our Privacy Policy

Reviewer #4: No

**********

[NOTE: If reviewer comments were submitted as an attachment file, they will be attached to this email and accessible via the submission site. Please log into your account, locate the manuscript record, and check for the action link "View Attachments". If this link does not appear, there are no attachment files.]

To ensure your figures meet our technical requirements, please review our figure guidelines: https://journals.plos.org/plosone/s/figures

You may also use PLOS’s free figure tool, NAAS, to help you prepare publication quality figures: https://journals.plos.org/plosone/s/figures#loc-tools-for-figure-preparation.

NAAS will assess whether your figures meet our technical requirements by comparing each figure against our figure specifications.

# Author comment: Author response to Decision Letter 2

**DOI:** 10.1371/journal.pone.0358972.r004

3 Jul 2026

We sincerely thank the Editor and the Reviewer for their valuable comments and constructive suggestions. We have carefully revised the manuscript to address all comments. A detailed point-by-point response has been provided in the attached Response to Reviewers document, and all changes have been incorporated into the revised manuscript.

Attachment Submitted filename: Response_to_Reviewers_auresp_2.docx

# Peer review: Decision Letter 2

**DOI:** 10.1371/journal.pone.0358972.r005

**Authors:** Vanessa Carels

9 Sep 2026

Statistical patterns in the Ethereum blockchain: analysis of EOAs and smart contracts in ERC20 token network

PONE-D-25-29678R2

Dear Dr. Saha,

We’re pleased to inform you that your manuscript has been judged scientifically suitable for publication and will be formally accepted for publication once it meets all outstanding technical requirements.

Within one week, you’ll receive an e-mail detailing the required amendments. When these have been addressed, you’ll receive a formal acceptance letter and your manuscript will be scheduled for publication.

An invoice will be generated when your article is formally accepted. Please note, if your institution has a publishing partnership with PLOS and your article meets the relevant criteria, all or part of your publication costs will be covered. Please make sure your user information is up-to-date by logging into Editorial Manager at Editorial Manager® and clicking the ‘Update My Information' link at the top of the page. For questions related to billing, please contact billing support.

If your institution or institutions have a press office, please notify them about your upcoming paper to help maximize its impact. If they’ll be preparing press materials, please inform our press team as soon as possible -- no later than 48 hours after receiving the formal acceptance. Your manuscript will remain under strict press embargo until 2 pm Eastern Time on the date of publication. For more information, please contact onepress@plos.org.

Kind regards,

Vanessa Carels

Staff Editor

PLOS One

Additional Editor Comments (optional):

Reviewers' comments:

Reviewer's Responses to Questions

**Comments to the Author**

Reviewer #4: All comments have been addressed

**********

2. Is the manuscript technically sound, and do the data support the conclusions?

Reviewer #4: Yes

**********

3. Has the statistical analysis been performed appropriately and rigorously?

Reviewer #4: Yes

**********

4. Have the authors made all data underlying the findings in their manuscript fully available?

The PLOS Data policy

Reviewer #4: Yes

**********

5. Is the manuscript presented in an intelligible fashion and written in standard English?

Reviewer #4: Yes

**********

Reviewer #4: (No Response)

**********

what does this mean?). If published, this will include your full peer review and any attached files.

If you choose “no”, your identity will remain anonymous but your review may still be made public.

**Do you want your identity to be public for this peer review?**  For information about this choice, including consent withdrawal, please see our Privacy Policy

Reviewer #4: No

**********

# Editor report: Acceptance letter

**DOI:** 10.1371/journal.pone.0358972.r006

**Authors:** Vanessa Carels

PONE-D-25-29678R2

PLOS One

Dear Dr. CHAKRABORTY,

I'm pleased to inform you that your manuscript has been deemed suitable for publication in PLOS One. Congratulations! Your manuscript is now being handed over to our production team.

At this stage, our production department will prepare your paper for publication. This includes ensuring the following:

* All references, tables, and figures are properly cited

* All relevant supporting information is included in the manuscript submission,

* There are no issues that prevent the paper from being properly typeset

You will receive further instructions from the production team, including instructions on how to review your proof when it is ready. Please keep in mind that we are working through a large volume of accepted articles, so please give us a few days to review your paper and let you know the next and final steps.

Lastly, if your institution or institutions have a press office, please let them know about your upcoming paper now to help maximize its impact. If they'll be preparing press materials, please inform our press team within the next 48 hours. Your manuscript will remain under strict press embargo until 2 pm Eastern Time on the date of publication. For more information, please contact onepress@plos.org.

You will receive an invoice from PLOS for your publication fee after your manuscript has reached the completed accept phase. If you receive an email requesting payment before acceptance or for any other service, this may be a phishing scheme. Learn how to identify phishing emails and protect your accounts at https://explore.plos.org/phishing.

If we can help with anything else, please email us at customercare@plos.org.

Thank you for submitting your work to PLOS One and supporting open access.

Kind regards,

PLOS One Editorial Office Staff

on behalf of

Dr. Vanessa Carels

Staff Editor

PLOS One
